# Extracellular Vesicles in Pathogenesis and Treatment of Metabolic Associated Fatty Liver Disease

**DOI:** 10.3389/fphys.2022.909518

**Published:** 2022-06-13

**Authors:** Ji Sun, Dianbao Zhang, Yiling Li

**Affiliations:** ^1^ Department of Gastroenterology, The First Affiliated Hospital of China Medical University, Shenyang, China; ^2^ Department of Stem Cells and Regenerative Medicine, Key Laboratory of Cell Biology, National Health Commission of China, and Key Laboratory of Medical Cell Biology, Ministry of Education of China, China Medical University, Shenyang, China

**Keywords:** extracellular vesicles, lipids, inflammation, pathogenesis, treatment, metabolic associated fatty liver disease

## Abstract

Metabolic associated fatty liver disease (MAFLD) is the most common chronic liver disease worldwide due to the sedentary and overeating lifestyle. Yet, the pathophysiology of MAFLD is still unclear and no drug has been approved for MAFLD treatment. Extracellular vesicles (EVs) are heterogenous membrane-bound particles released from almost all types of cells. These nano-sized particles mediate intercellular communication through their bioactive cargos including nucleic acids, proteins, and lipids. The EVs modulate metabolic homeostasis *via* communication between adipose tissue and liver. The dysregulation of lipid metabolism leads to inflammation in liver and the number and compounds of EVs are changed during MAFLD. The injured hepatocytes secrete EVs to induce the migration of bone marrow-derived monocytes and the activation of macrophages in liver. The EVs secreted by different cells regulate the alteration of hepatic stellate cell (HSC) phenotypes and HSC activation gives rise to liver fibrosis. Based on the participation of EVs in MAFLD progression, we discuss the prospects of EVs as a therapeutic target and their application in drug delivery.

## Introduction

Non-alcoholic fatty liver disease (NAFLD), the most common chronic liver disease, affects about 25 percent of the world’s population and causes a heavy economic burden (Younossi et al., 2016). In China, the prevalence of NAFLD is about 30% (Wu et al., 2020a; Wang et al., 2021), and the number of patients is predicted to reach 314.58 million in 2030 (Estes et al., 2018). Previously, NAFLD was mainly characterized by steatosis in >5% hepatocytes, without other fat accumulation reasons such as alcohol consumption, steatogenic medication use and hereditary diseases (Chalasani et al., 2012; European Association for the Study of the Liver (EASL)European Association for the Study of Diabetes (EASD)European Association for the Study of Obesity (EASO), 2016). But, NAFLD can coexist with viral hepatitis, autoimmune liver disease and alcoholic hepatitis. Furthermore, alcohol intake reported by patients is often inaccurate in clinical practice. These issues challenge the definition and diagnosis of NAFLD. Recently, an international consensus panel on fatty liver renamed this disease from NAFLD to metabolic associated fatty liver disease (MAFLD) (Eslam et al., 2020a). The new diagnosis of MAFLD is based on liver steatosis confirmed by liver biopsy, imaging method, or blood markers, with one of the following conditions: overweight/obesity, type 2 diabetes mellitus (T2DM), or metabolic syndrome (Eslam et al., 2020b). Formerly, the spectrum of NAFLD included simple steatosis, NAFLD associated steatohepatitis (NASH) and advanced fibrosis, cirrhosis, or even hepatocellular carcinoma (Younossi et al., 2019). But in new criteria, the spectrum of disease is not divided into “NASH” and “not-NASH.” MAFLD is used as a single overarching term to reflect the continuous disease progression (Eslam et al., 2020b). The pathophysiology underlying MAFLD is highly intricate. It evolved from the “two-hit” hypothesis to the “multiple parallel hits” theory (Tilg and Moschen, 2010), and the alteration of denomination indicated that MAFLD was a feature of metabolic syndrome (Marchesini et al., 2001; Eslam et al., 2020a). Novel regulatory molecules and pathways are being discovered, but associated pharmaceutical therapies have not been approved.

Extracellular vesicles (EVs) are a heterogeneous collection of nano-sized vesicles, involving exosomes, microvesicles and apoptotic bodies. They contain various surface markers and cargos derived from parental cells. The surface molecules on EVs can bind to the receptors on recipient cells, inducing intracellular signaling pathways. Then, EVs are internalized *via* endocytosis, phagocytosis or directly fusing with the membrane of target cells (Devhare and Ray, 2018; van Niel et al., 2018). In normal conditions, cells release EVs to exchange messages. Pathological conditions change the biogenesis mechanism and contents of EVs. Besides, EVs can be artificially modified by engineering surface molecules or loading therapeutic agents (Tang et al., 2020). The exciting features of EVs intrigue us to study the roles of EVs in the pathophysiology and treatment of MAFLD.

## What are Extracellular Vesicles?

### Definition and Classification of Extracellular Vesicles

EVs, nanoparticles with lipid bilayers and cytosolic components, are released into extracellular space by virtually all active cells. The cargos of EVs comprise nucleic acids (mRNA, microRNA, long non-coding RNA, genomic DNA, or mitochondrial DNA), proteins and lipids. The lipid bilayers of EVs protect these compounds from degradation in the extracellular environment (Rilla et al., 2019). EVs are ubiquitously present in human body fluids, including plasma, urine, bile, breast milk, saliva, semen, synovial fluid, ascites, cerebrospinal fluid, pleural effusions, bronchoalveolar lavage fluids, and even feces (Witwer et al., 2013). Also, EVs could be collected from culture supernatants of many eukaryotic cells (Giebel and Helmbrecht, 2017). According to their size, surface markers and biogenesis, EVs are classified into three subcategories (Table 1).

**TABLE 1:** Three subcategories of EVs.

Characteristic	Exosomes	Microvesicles	Apoptotic bodies
Size	30–250 nm Rilla et al. (2019)	100–1000 nm Rilla et al. (2019)	1–5 μm Rilla et al. (2019)
Formation	Release after multivesicular bodies fuse with plasma membrane	Budding of plasma membrane	Release after cell apoptosis
Surface markers	Tetraspanins (CD9, CD63, CD37, CD81, CD82) Rilla et al. (2019); ESCRT proteins (Alix, TSG101); heat shock proteins (HSP70, HSP90) Ban et al. (2016); Andreu and Yáñez-Mó, (2014); GM1ganglioside Balaphas et al. (2019)	Phosphatidylserine (PS) Balaphas et al. (2019); cholesterol Balaphas et al. (2019)	Not applicable

### Biogenesis and Release of Extracellular Vesicles

**FIGURE 1:**
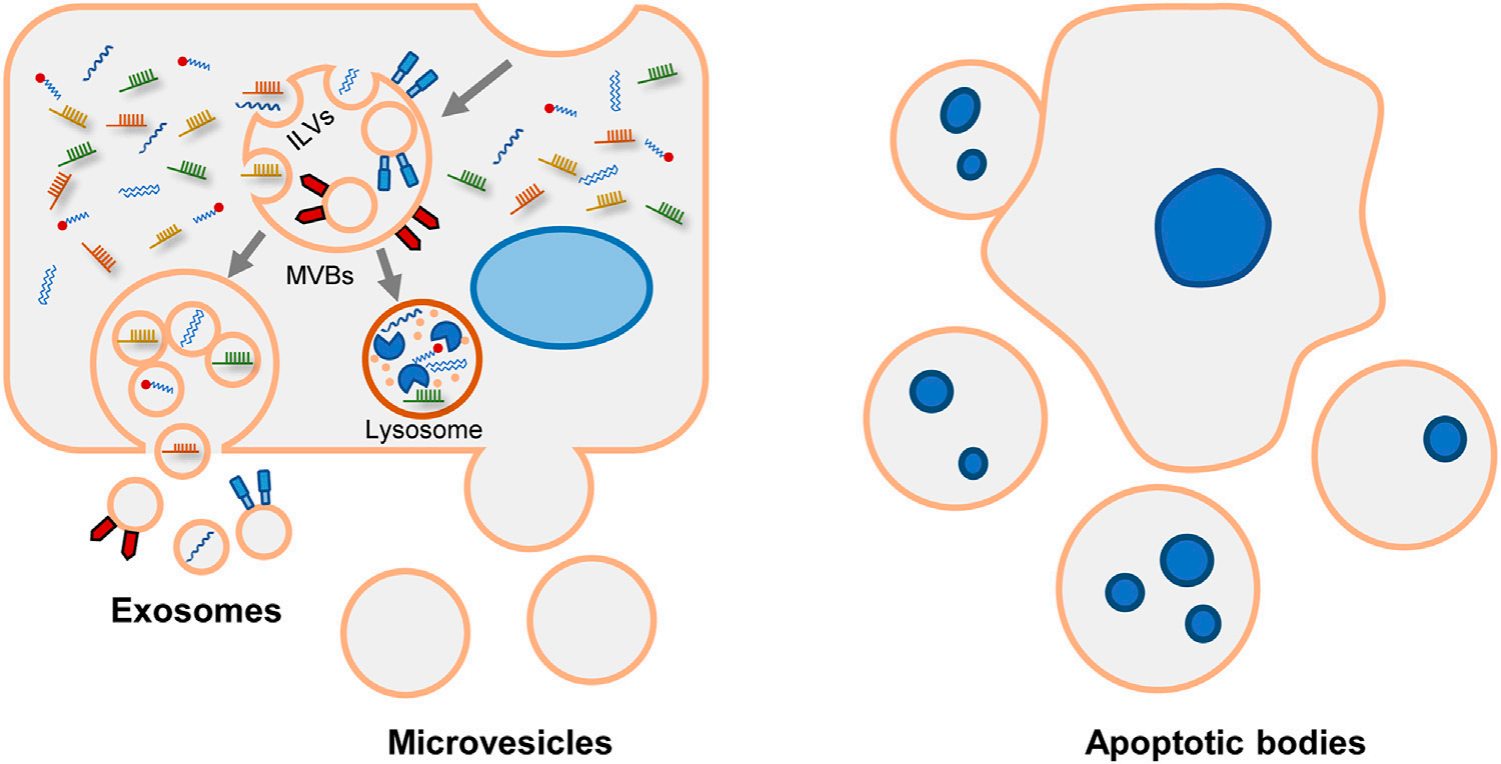
Biogenesis and release of various EVs. Generally, EVs are divided into exosomes, microvesicles and apoptotic bodies. Exosomal formation begins with endocytic process. After the endosomal membrane invagination, intraluminal vesicles (ILVs) are formed. The endosomes containing ILVs are referred as multivesicular bodies (MVBs). Subsequently, MVBs have two fates. They can be degraded in lysosomes. Alternatively, MVBs merge with plasma membrane and release ILVs as exosomes. Microvesicles are formed through direct budding process. Apoptotic bodies are formed during apoptosis.

In response to various stimuli and stress, cells secrete EVs *via* different mechanisms (Figure 1). Exosome is a class of EVs studied extensively. During the endocytosis process, the cell membrane invaginates, and then early endosomes are formed. As intraluminal vesicles (ILVs) bud into endosome membrane, the early endosomes mature into late endosomes which are referred as multivesicular bodies (MVBs). Subsequently, MVBs degrade in lysosomes or fuse with the plasma membrane to release ILVs as exosomes (Xu et al., 2018; Rilla et al., 2019).

The mechanisms of ILVs assembly and MVBs formation are complicated. The endosomal sorting complex required for transport (ESCRT) is the central machinery that determines cargo sorting, cargo clustering, and the invagination of endosomes (Hirsova et al., 2016a). Alternatively, some ESCRT-independent pathways have been described, including neutral sphingomyelinase (nSMAse-2), ceramide (Trajkovic et al., 2008; Bianco et al., 2009), tetraspanins CD9, and CD63 (Andreu and Yáñez-Mó, 2014). Besides, syntenin modulates the intraluminal budding of endosomal membranes. In time of ILVs formation, syntenin binds to syndecan and recruits ALIX, an accessory protein of ESCRT (Baietti et al., 2012). Heparanase trims the heparan sulfate chains on syndecan and activates the syndecan-syntenin-ALIX pathway (Roucourt et al., 2015). Syntenin-mediated exosomes biogenesis also depends on the small GTPase ADP ribosylation factor 6 (ARF6) and its effector phospholipase D2 (PLD2) (Ghossoub et al., 2014). And tyrosine kinase SRC is the upstream regulator of ARF6/PLD2 (Imjeti et al., 2017). The mechanisms vary among different cell types. For instance, CXCR1 and CXCR2 coordinate the release of exosomes in hepatocytes (Lee et al., 2021); adiponectin increases exosomes formation in MVBs in endothelial cells (Obata et al., 2018).

**FIGURE 2:**
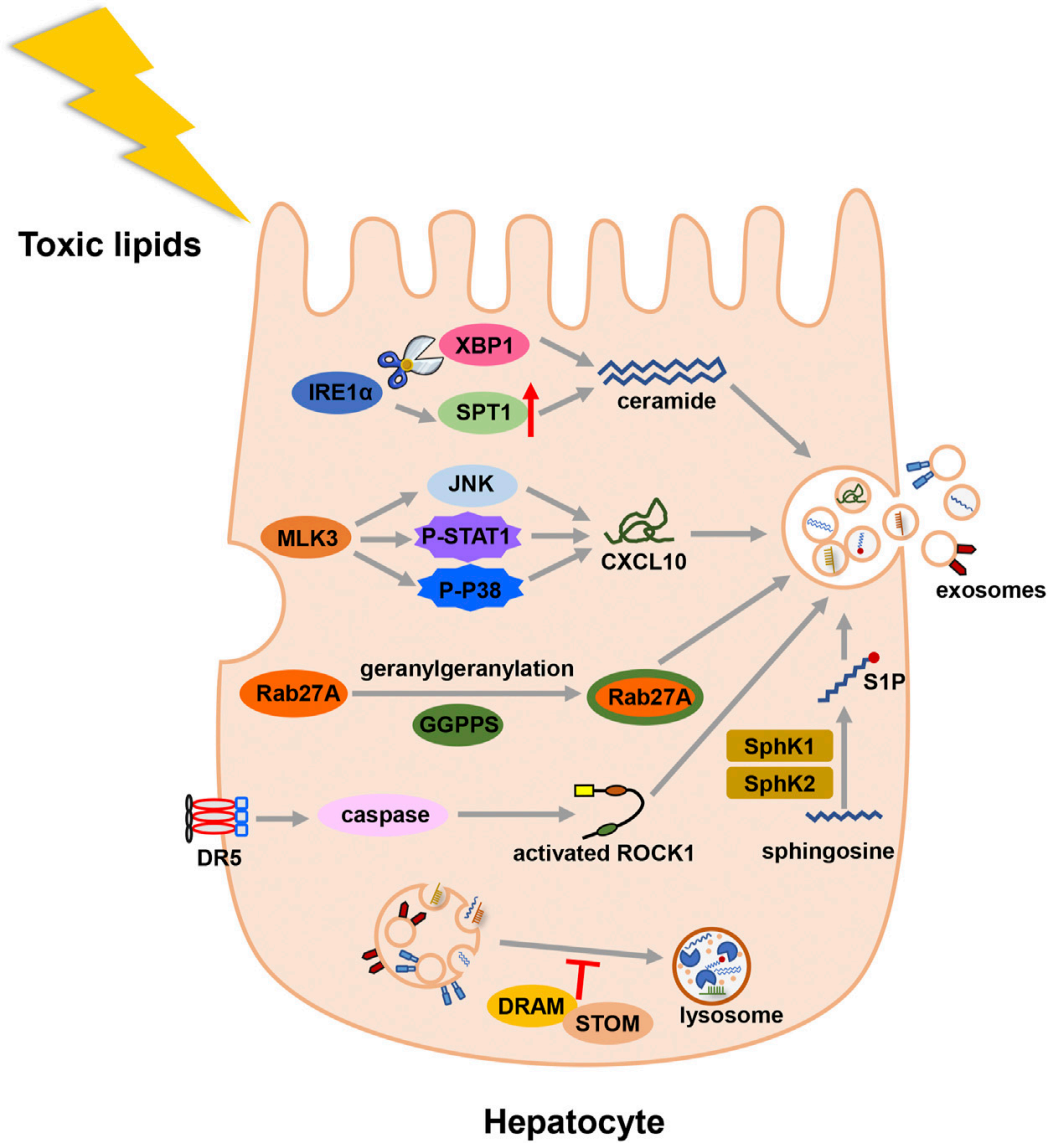
Illustration for the biogenesis fo exosomes in MAFLD. Toxic lipid accumulation is the major hit of MAFLD, which causes more secretion of exosomes by multiple mechanisms. Abbreviation: death receptor 5 (DR5), Rho-associated kinase 1 (ROCK1), geranylgeranyl diphosphate synthase (Ggpps), mixed lineage kinase 3 (MLK3), (C-X-C motif) ligand 10 (CXCL10), inositol requiring enzyme 1α (IRE1α), X-box binding protein-1 (XBP1), serine palmitoyltransferase1 (SPT1), sphingosine-1-phosphate (S1P), sphingosine kinase 1 (SphK1), and sphingosine kinase 2 (SphK2), damage-regulated autophagy modulator (DRAM), stomatin (STOM).

Different external stimuli also affect the formation of exosomes. In MAFLD state, toxic lipids are often accumulated in hepatocytes and regulate the formation of exosomes (Figure 2). Study showed that saturated free fatty acids activated death receptor 5 (DR5) and recruited caspase 8 and caspase 3 (Cazanave et al., 2011). Then caspase 3 cleaved and activated Rho-associated kinase 1 (ROCK1) (Coleman et al., 2001). And the release of hepatic exosomes was mediated by this signaling pathway: DR5 → caspase 8 → caspase 3 → ROCK1 (Hirsova et al., 2016b). During the early stage of lipid accumulation, geranylgeranyl diphosphate synthase (Ggpps) was upregulated and induced the secretion of exosomes in hepatocytes. In details, Ggpps geranylgeranylated a Rab GTPase-Rab27A and promoted MVBs distribution and exosome formation (Zhao et al., 2020). Besides, mixed lineage kinase 3 (MLK3) activated JNK and aggravated MAFLD progression (Ibrahim et al., 2014). The secretion of exosome during MAFLD was also regulated by MLK3 and activated JNK. Treated with MLK3 inhibitor or JNK inhibitor, hepatocytes released less exosomes. Subsequently, exosomes were loaded with a specific cargo-(C-X-C motif) ligand 10 (CXCL10), which depended on P-STAT1 and P-P38 phosphorylated by MLK3 (Ibrahim et al., 2016). As a sensor of lipotoxic stress, inositol requiring enzyme 1α (IRE1α) could increase the release of exosomes by splicing X-box binding protein-1 (XBP1). Then, IRE1α packaged ceramide *via* upregulating serine palmitoyltransferase1 (SPT1) (Kakazu et al., 2016a). In addition to ceramide, sphingosine-1-phosphate (S1P) was enclosed in exosomes of hepatocytes (Kakazu et al., 2016a). Another study found sphingosine kinase 1 (SphK1) and sphingosine kinase 2 (SphK2) induced the release of exosomes in lipotoxic hepatocytes by mediating the formation of S1P (Liao et al., 2018). Lysosome can degrade MVBs before MVBs fuse with the cell membrane. In MAFLD patients, damage-regulated autophagy modulator (DRAM) could recruit stomatin (STOM) and increase the lysosomal membrane permeabilization. MVBs were degraded less and released more exosomes (Zhang et al., 2021).

Unlike exosomes, microvesicles and apoptotic bodies are formed *via* direct exocytosis. The initiate process of exocytosis depends on the contraction of actin-myosin. Phosphatidylserine (PS) is usually segregated in the inner layer. Whereas in response to stimulation, PS is externalized *via* the activation of flippases and floppases, and causing MVs release. So, it allows PS to be a characteristic lipoprotein of microvesicles (Hugel et al., 2005). But not all microvesicles express PS. For example, partial platelet-derived microvesicles are PS-negative (Arraud et al., 2014; Lundström et al., 2020). The overlapped size and the limitation of purification technology make it challenging to categorize exosomes or microvesicles accurately (Xu et al., 2015; Giebel and Helmbrecht, 2017; Chuo et al., 2018; Nolan and Duggan, 2018). In this review, EVs are used as a general term to represent exosomes and microvesicles. Apoptotic bodies are not the research focus of EVs, so they are not discussed.

## Extracellular Vesicles and Metabolic Associated Fatty Liver Disease Pathogenesis

With the in-depth study, the “two-hit” hypothesis of MAFLD is challenged. To supplement the deficiency of previous theory, the “multiple parallel hits” theory has been proposed, which involves lipotoxicity, adipokines, insulin resistance (IR), intestinal dysfunction, endoplasmic reticulum (ER) stress, diet, and genetic factors (Tilg and Moschen, 2010; Noureddin and Sanyal, 2018; Tilg et al., 2021). Recent studies confirmed that primary hepatocytes (Conde-Vancells et al., 2008; Ogese et al., 2019), hepatocellular carcinoma cells (Matilainen et al., 2020), hepatic stellate cells (Kostallari et al., 2018), adipocytes (Crewe and Scherer, 2021) and liver sinusoidal endothelial cells (Wang et al., 2015) secreted EVs. A proteomic analysis of nontumoral hepatocytes-derived EVs identified 244 proteins in EVs. Gene ontology (GO) analysis revealed that these proteins had relation to intracellular transport, lipid metabolism, catabolic process and the generation of precursor metabolites and energy (Conde-Vancells et al., 2008). In MAFLD mice models, Povero et al. observed early increased release of EVs in the liver and blood (Povero et al., 2014). And there was a correlation between the elevated level of EVs and hepatocyte cell death, fibrosis, and pathological angiogenesis. In human bodies, Kornek et al. found circulating EVs derived from immune cells were elevated in patients with MAFLD versus healthy individuals. And EVs number also correlated with the severity of MAFLD (Kornek et al., 2012). Here, we reviewed recent findings and discussed how EVs participated in the different stages of MAFLD.

### Extracellular Vesicle: A Mediator of Lipid Metabolic Dysfunction

MAFLD can be considered as the hepatic manifestation of systematic metabolic disorder. Insulin pathways involve closely in metabolism homeostasis. When insulin response is diminished, or the insulin pathway is impaired, IR occurs, which contributes to the onset and progression of MAFLD (Khan et al., 2019; Gerges et al., 2021; Rinaldi et al., 2021). In a rat model of MAFLD, insulin associated PI3K/Akt/mTOR pathway was inhibited (Sztolsztener et al., 2021). In obese people preexisting T2DM, the hyperinsulinemia and lower insulin sensitivity was related to the presence of MAFLD (Lomonaco et al., 2016). Compared with low IR people, high IR people had higher hepatic steatosis, and inflammation (Luukkonen et al., 2016). Besides, insulin resistance index (HOMA-IR) could be a diagnostic indicator of MAFLD to some degree (Isokuortti et al., 2017). Adipose tissue plays important roles in the development of MAFLD (Azzu et al., 2020). Several studies have shown that adipose tissue can function as an endocrine organ and secrete EVs to regulate IR of hepatocytes. In obesity mice, adipose tissue-derived EVs contained less miR-141-3p and impeded insulin response of hepatocytes (Dang et al., 2019). Furthermore, a study of human adipose tissue showed that most of EVs inhibited insulin signaling. And many adipokines existed in EVs, such as monocyte chemoattractant protein-1 (MCP-1), migration inhibitory factor (MIF) and interleukin-6 (IL-6) (Kranendonk et al., 2014). Obesity also results in the accumulation of proinflammatory macrophages in adipose (Weisberg et al., 2003). These macrophages secreted EVs, which contained miR-155 and miR-29a, and depressed insulin sensitivity of hepatocytes (Ying et al., 2017; Liu et al., 2019).

In MAFLD patients, IR stimulated *de novo* lipogenesis of hepatocytes (Smith et al., 2020). So beyond impairing insulin signaling, EVs from adipocytes also induced following fatty accumulation. Li et al. reported that adipocytes-secreted EVs could deliver miR-199a-5p and foster hepatic lipid deposition and steatosis (Li et al., 2020). The function of miR-199a-5p was achieved *via* targeting mammalian sterile 20-like kinase 1 (MST1) and the downstream SREBP-1c and AMPK pathway. In adipocytes, AMPKα1 was found to impede EVs secretion (Yan et al., 2021). EVs shed from AMPKα1 deficient adipose tissue elevated the level of triglycerides (TGs), cholesterol in liver. In these EVs, CD36, a fatty acid transporter (Rada et al., 2020), had high level among 43 different protein cargos. CD36 promoted the internalization of EVs and aggravated the lipid accumulation and apoptosis of HepG2 cells. Metformin, which relieved diabetes *via* the activation of AMPK, was also found to suppress the secretion of EVs and alleviate high fat diet (HFD)-induced fatty liver (Yan et al., 2021). Under ER stress, adipocytes secreted more EVs, which encapsulated aldo-keto-reductase 1B7 (Akr1b7). After targeting hepatocytes, Akr1b7 could augment the accumulation of glycerol and TGs and elevated inflammation factors and fibrosis indicators (Gu et al., 2021). Another molecule involved in adipose tissue-liver crosstalk was resistin, which was rich in adipocytes-generated EVs (Rong et al., 2019). Injecting resistin-enriched EVs elevated the serum TGs and hepatic lipid accumulation of mice. But interestingly, the markers associated with inflammation, fibrosis and cell apoptosis were not obviously altered in hepatocytes. This meant resistin-enriched EVs might trigger hepatic steatosis without eliciting hepatic inflammation. Melatonin was found to reduce the level of resistin in EVs and alleviate the HFD-induced hepatic steatosis.

**FIGURE 3:**
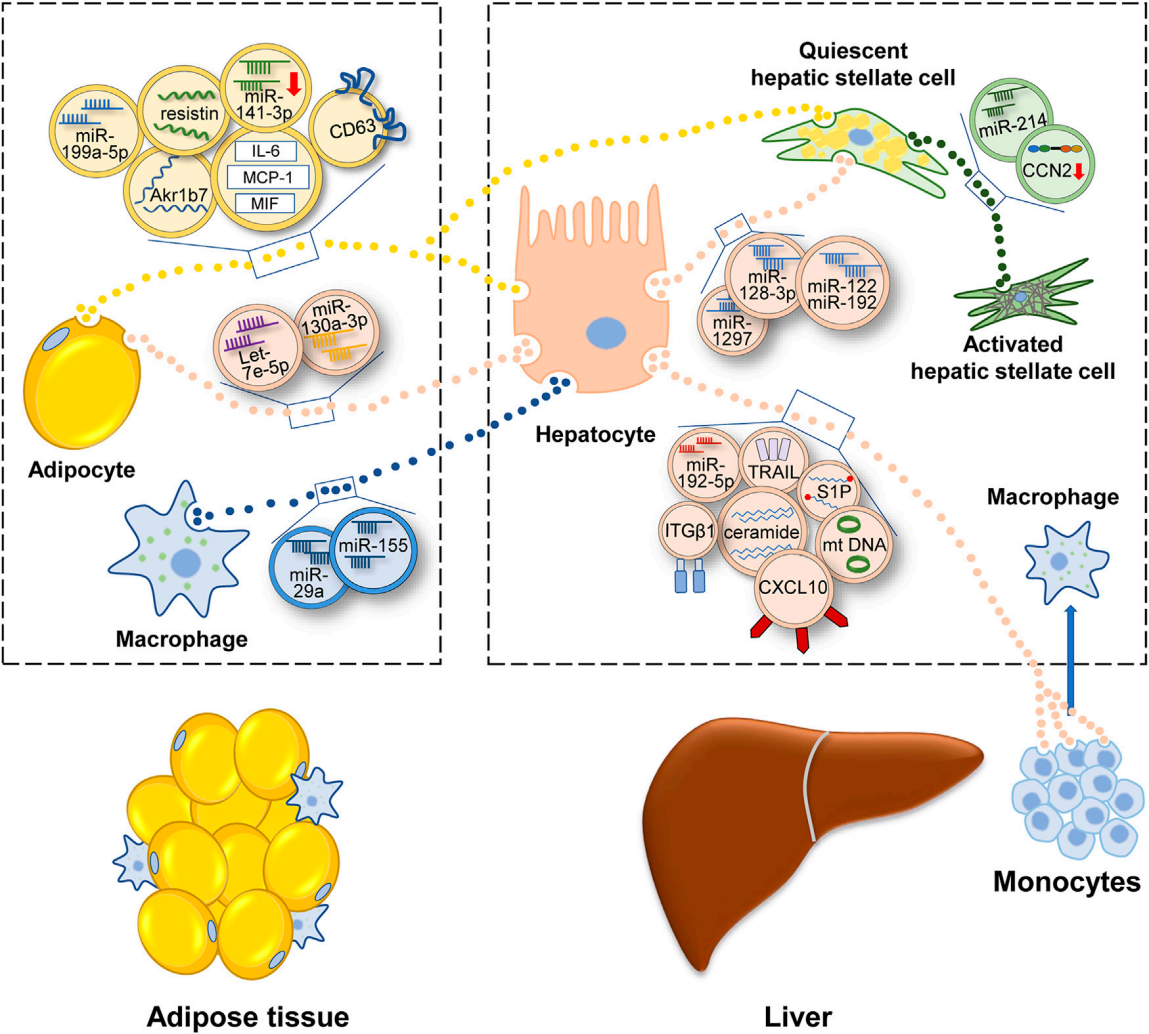
Illustration for how EVs participate in the development of MAFLD. Under the condition of metabolic disturbance, cells secrete EVs to transfer information, which contain protein, lipid, miRNA, lncRNA or DNA. Adipocytes and macrophages in adipose tissue transfer EVs to liver and regulate the lipid metabolism and fibrotic phenotype in liver. Inside liver, EVs link up the communication of various hepatic cells and promote MAFLD progression. EVs secreted outside the liver can be consumed by adipocytes and monocytes, then regulating energy metabolism and inflammatory condition. Abbreviation: aldo-keto-reductase 1B7 (Akr1b7), cluster of differentiation 36 (CD36), interleukin- 6 (IL-6), monocyte chemoattractant protein-1 (MCP-1), migration inhibitory factor (MIF), connective tissue growth factor (CCN2), mitochondrial DNA (mtDNA), sphingosine-1-phosphate (S1P), tumor necrosis factor-related apoptosis-inducing ligand (TRAIL), integrin β1(ITGβ1), C-X-C motif ligand 10 (CXCL10).

Above studies suggested that adipose tissue-derived EVs probably were “harmful,” especially in the obese state. Liver was the major target of adipose tissue-derived EVs. And the final effect included IR, lipid accumulation, inflammatory response, and fibrosis progression (Figure 3). Classical drugs, metformin and melatonin might relieve MAFLD *via* inhibiting the release of “harmful” EVs or reducing the “harmful” cargos levels in adipocyte-derived exosomes.

The lipid dynamics in liver can be charactered by adipose EVs. Meanwhile, hepatocytes also secrete EVs to regulate adipogenesis. In HFD-fed mice, hepatic EVs increased lipid deposition of adipocytes. Detecting the contents of EVs, let-7e-5p had the highest level. It was found that let-7e-5p could target a metabolic gene PPARγ coactivator-1 *α* (Pgc1α) and accelerate lipogenesis and impede lipid oxidation of adipocytes (Zhao et al., 2020).

As a response to the cellular damage, adipose tissue and liver secrete EVs and interact lipid metabolism. As to the exact role of EVs, it still needs further study. In future researches, we’d better increase the sample volume and incorporate different adipose tissue, such as epididymal, visceral and retroperitoneal adipose tissue into studies. The progressive dysfunction of adipose tissue is the key event of MAFLD. How liver monitor the adipose metabolism by EVs merits more researches.

### Extracellular Vesicles: Linkage Between Liver and Inflammatory Cells

The progression of MAFLD is accompanied by the inflammatory response. The hallmarks of activation of the immune system include polarization of Kupffer cells (KCs) and recruitment of bone marrow-derived monocytes. Previous studies revealed that HFD and free fatty acids (FFAs) could activate KCs *in vivo* and *in vitro* (Xue et al., 2014; Duarte et al., 2015). Recent studies implied that EVs might be the intermediary agent of inflammatory response (Figure 3). Deng et al. (2009) reinjected EVs of HFD-fed mice into regular chow diet-fed mice. It was found that immature myeloid cells accumulated in liver and the levels of IL-6, aspartate aminotransferase (AST) and alanine aminotransferase (ALT) in serum were elevated (Deng et al., 2009). Whereas, a similar experiment conducted in humans had a contrary result. Circulating EVs isolated from the plasma of normal or obese people intended to impede inflammation in HepG2 cells, evidenced by the declining level of IL-6 and TNF-α (Afrisham et al., 2021). The opposite conclusion still needs further proof because of the small number of study samples. In addition, both two studies did not demonstrate the cell sources of EVs directly. Another study may be more accurate. Garcia-Martinez et al. (2016) isolated EVs from the serum of mice and patients with steatohepatitis and verified that the source of EVs was hepatocyte. These hepatic EVs contained oxidized mitochondrial DNA and recruited inflammatory cells depending on the activation of toll-like receptor (TLR) (Garcia-Martinez et al., 2016).

The accumulation of harmful lipid droplets, such as FFAs, TGs, free cholesterol, lysophosphatidyl choline (LPC), bile acids, and ceramides induces lipotoxicity in liver (Mendez-Sanchez et al., 2018). Many cells in the liver are affected by lipotoxicity, including hepatocytes, stellate cells, macrophages, and bile duct cells (Marra and Svegliati-Baroni, 2018). Palmitate acid (PA), a kind of FFAs, enabled hepatic EVs to recruit bone marrow-derived macrophages (BMDMs). Interestingly, these PA-induced EVs contained a common lipid: ceramide. And IRE1α, which facilitated EVs secretion, was the key director for macrophages accumulation in liver (Kakazu et al., 2016b; Dasgupta et al., 2020). In another study, cutting down the level of S1P in EVs inhibited the chemotaxis of macrophages. Thus, S1P in lipotoxic hepatocytes may also influence macrophages (Liao et al., 2018).

In addition to these bioactive lipid species, EVs also encapsulate a diverse range of cargos, such as signaling proteins, oxidized molecules or miRNAs so as to transmit proinflammatory information (Ipsen and Tveden-Nyborg, 2021). Ibrahim et al. (2016) found that LPC-treated hepatocytes secreted more EVs, which were loaded with CXCL10. Then these EVs activated macrophages by CXCL10-CXCR3 interaction (Ibrahim et al., 2016). Another study elucidated that tumor necrosis factor-related apoptosis-inducing ligand (TRAIL), a membrane protein of lipotoxic EVs, activated macrophage *via* the interplay with DR5 (Hirsova et al., 2016b). Similarly, LPC activated integrin β1 (ITGβ1) in hepatocytes and cytoplasmic ITGβ1 was shipped into EVs. Then these EVs were released into the circulation and fostered BMDMs adhering to liver sinusoidal endothelial cells (LSECs) by the binding between ITGβ1 and vascular cell adhesion molecule 1 (VCAM-1) (Guo et al., 2019). In another recent study, miR-192-5p in lipotoxic hepatocyte-derived EVs activated macrophages by regulating the Rictor/Akt/FoxO1 signaling cascade (Liu et al., 2020).

Collectively, these evidences suggested the important role of EVs in converting dysfunctional metabolic signal into proinflammatory signal and inducing inflammation signaling cascade. Other immune cells also play a crucial part in MAFLD, such as, neutrophils, T-helper (Th), and cytotoxic CD8^+^ T cells (Kuchay et al., 2020). There is still a broad space to investigate how EVs regulate immune cells of MAFLD patients.

### Extracellular Vesicles Targeting Hepatic Stellate Cell Fuel Fibrosis in Metabolic Associated Fatty Liver Disease

As a major determinant of poor outcome in MAFLD progression, liver fibrosis is characterized by an unbalanced restoration of extracellular matrix (ECM). The key node of hepatic fibrogenesis is the activation of hepatic stellate cells (HSCs). Then HSCs transdifferentiate into myofibroblasts-like cell type (Tsuchida and Friedman, 2017).

Many liver cells can activate HSCs, involving hepatocytes, endothelial cells (Wang et al., 2015), natural killer (NK) cells (Wang et al., 2020) or macrophages. They constitute a cellular network, which is connected by EVs (Figure 3). In this section, we center on those researches whose models are relevant to MAFLD. For example, EVs isolated from lipotoxic hepatocytes leaded to a phenotype switch of HSCs from quiescence to activation. MiR-128-3p was rich in these EVs and targeted PPARγ (Povero et al., 2015). Using a microarray, Lee et al. (2017) found that lipotoxic hepatocytes-derived EVs had a remarkably increased level of miR-122 and miR-192, which were extensively reported in the development of MAFLD (Lee et al., 2017; Khalifa et al., 2020). MiR-192 upregulated the expression of fibrosis markers and activated the profibrogenic function of HSCs. And elevated miR-122 and miR-192 were also observed in patients with advanced-stage MAFLD compared to those with early-stage MAFLD (Lee et al., 2017). Recently, miRNA-1297 in lipotoxic hepatocytes-derived EVs was identified to activate HSCs *via* PTEN pathway (Luo et al., 2021a). In addition to the treatment with excess fatty acid, obstructive sleep apnea syndrome (OSAS) also contributed to steatohepatitis and fibrosis (Nobili et al., 2014). Hepatocytes under chemic hypoxia were detected to secret EVs, which caused increased expression of profibrotic cytokines in HSCs (Hernández et al., 2020). Besides, HSCs can be awakened by EVs secreted from activated HSCs (Charrier et al., 2014). EVs delivered connective tissue growth factor (CCN2) to other quiescent or activated HSCs and upregulated the expression of fibrotic genes. MiR-214 that regulated CCN2 negatively was also identified in activated HSCs-derived EVs. Correspondingly, the level of miR-214 in EVs was low (Chen et al., 2014). As an endocrine organ, adipose tissue also could trigger fibrosis *via* delivering EVs into HSCs and modulate transforming growth factor beta (TGF-β) pathway (Koeck et al., 2014).

## Extracellular Vesicles: Potential Therapeutical Tools in Metabolic Associated Fatty Liver Disease

The severity of MAFLD depends on the grade of activity and fibrosis progression. A scoring system, NAFLD activity score (NAS), quantifies the activity of MAFLD, including steatosis, inflammation, and hepatocellular ballooning. Recent medical studies confirmed that lifestyle modification could degrade NAS and alleviate the early stage of fibrosis (Romero-Gómez et al., 2017). The underlying mechanism of exercise in systemic improvement is still unclear. Probably, this beneficial effect was modulated by EVs (Safdar et al., 2016). During endurance exercise, the number of EVs was elevated and their cargos, such as proteins and miRNAs were also changed (Estébanez et al., 2021). Then exercise-derived EVs were intended to localize in liver and exchange their compounds (Whitham et al., 2018).

Regardless of the mechanism, it is definite that physical exercise is a primary therapy for MAFLD. But in terms of medical treatment, there has been no breakthrough in drug research. As discussed before, EVs participate in the development of MAFLD. These “harmful” EVs can amplify fat accumulation, promote the polarization and infiltration of immune cells, and activate HSCs. So, it is plausible to attenuate MAFLD by inhibiting the formation, cargo sorting and release of EVs (EL Andaloussi et al., 2013). For example, it turned out that the release of EVs from lipotoxic hepatocytes depended on ROCK1. The injection of ROCK1 inhibitor fasudil decreased the serum EVs of MAFLD mice and ameliorated the severity of MAFLD (Hirsova et al., 2016b). Pharmacological inhibition of MLK3 was found to impede the enrichment of CXCL10 in EVs and alleviate MAFLD progression in mice (Ibrahim et al., 2016; Tomita et al., 2017). Fat, fructose, and cholesterol (FFC) diet-induced steatohepatitis could be attenuated by pharmacologic IRE1α inhibition and genetic IRE1α knockout (Dasgupta et al., 2020). But proteins that modulate the biogenesis of EVs also affect other vital cellular functions, such as endolysosomal trafficking (Hirsova et al., 2016a). So, how to precisely regulate target effects is the important direction of future research.

During the progression of MAFLD, EVs played a “harmful” role in the crosstalk between adipose tissue and liver. However, during experimental stem cell therapy, stem cells-derived EVs may serve as a “salutary” medium to halt the inflammation and fibrosis of MAFLD. For instance, both human adipose tissue-derived mesenchymal stem cells (AD-MSCs) and their EVs reduced the level of ALT and liver fibrosis in MAFLD mice. Meanwhile, it was observed that anti-inflammatory macrophages were increased in liver (Watanabe et al., 2020). Similarly, another study confirmed that AD-MSCs and their EVs attenuated hepatic steatosis through the interaction with macrophages. AD-MSCs-derived EVs delivered signal transducer and activator of transcription3 (STAT3) into macrophages, and shifted them into anti-inflammation M2 phenotype (Zhao et al., 2018).

Besides adipose tissue, mesenchymal stem cells (MSCs) from other sources also released EVs and exerted protective effect in MAFLD. Ohara et al. (2018) injected amnion-derived MSCs (AMSCs)-derived EVs into steatohepatitis mice. Then the inflammation and fibrosis were alleviated perhaps *via* inactivating Kupffer cells and HSCs (Ohara et al., 2018). Likewise, induced pluripotent stem cells (iPSCs)-derived EVs downregulated the expression of fibrotic genes and inhibited the proliferation and chemotaxis of HSCs. In miRNA profile of iPSC-derived EVs, miR-92a-3p had a high expression level (Povero et al., 2019). A recent study reported that human liver stem cells (HLSCs)-originated EVs improved the inflammation and fibrosis of MAFLD (Bruno et al., 2020a). Notably, after proteomic analysis, several anti-inflammation proteins were enriched in HLSCs-originated EVs. Therefore, it was speculated that these bioactive cargos might serve as the actual mediators.

Compared with conventional drug delivery platforms, EVs have unique superiorities, such as low immunogenicity, less tumorigenicity, biological barriers penetrability, biocompatibility, and less exogenous infection (Bruno et al., 2020b; Hu et al., 2020; Tang et al., 2020). Thus, it is possible to use EVs to transport exogenous molecules. With the structure of hydrophobic lipid membrane and an aqueous core, EVs may address the shortcomings of RNA-based therapies, such as degradation, low uptake, and off-target effect (Wiklander et al., 2019). Qu et al. (2017) transfected pre-miR-181-5p into AD-MSCs and collected EVs with an enrichment of miR-181-5p (Qu et al., 2017). These EVs repressed HSCs activation and induced HSCs autophagy *via* STAT3/Bcl-2/Beclin1 signaling pathway. Besides, liver fibrosis could be attenuated by EVs *in vitro* and *in vivo*. Similarly, AD-MSCs-derived EVs loaded with miR-122 showed therapeutic potential in liver fibrosis (Lou et al., 2017). MiR-130a was decreased in the liver of MAFLD rats and in the islet of non-obese spontaneous type 2 diabetes, indicating that it might be protective for metabolic disorder (Jin et al., 2009; Esguerra et al., 2011; Xiao et al., 2014). Using CRISPR/CAS9 technique and mimic transfection method, the model of miR-130a overexpressed hepatocytes was constructed *in vivo* and *in vitro*. EVs released from these hepatocytes contained more miR-130a. It was found that these hepatic EVs suppressed fat synthesis and ameliorated IR of adipocytes. And miR-130a could target PH domain leucine-rich repeat protein phosphatase 2 (PHLPP-2) and AKT-AS160-GLUT4 pathway (Wu et al., 2020b). As we can see, after transfection, parental cells can deliver EVs which contain therapeutic miRNAs. In addition to transfection, Momen-Heravi et al. (2014) provided a novel sight to load EVs with exogenous miRNAs (Momen-Heravi et al., 2014). Using electroporation, EVs were rich in miR-155 mimics and could replenish the hepatic level of miR-155 in miR-155 knockout mice. Additionally, the delivery of miR-155 by EVs was more efficient and less cytotoxic than classical transfection. A recent study even applied gene therapy to EVs delivery (Luo et al., 2021b). After transfection, the clustered regularly interspaced short palindromic repeats (CRISPR)/CRISPR-associated protein 9 (dCas9)-VP64 system could be detected in mice hepatocytes-derived EVs. Then CRISPR/dCas9-VP64 system activated hepatocyte nuclear factor 4a (HNF4α) gene in HSCs and improved fibrosis.

The therapeutic applications of EVs include regulating the formation of EVs and employing EVs as a neo-drug delivery system (Tang et al., 2020). Preclinical studies of MSCs-EVs showed their promising properties in reducing fibrosis and counteracting inflammation. Most studies of MSC-EVs therapy were conducted by direct injection *in vivo* or co-cultivation *in vitro*. Future studies can pay more attention to the catalogs and levels of cargos and the mechanisms in modulating target cells. According to the existing reports and www.clinicaltrials.gov, EVs-mediated MAFLD therapy has not yet been tested in clinical study (Massa et al., 2020). Meanwhile a set of challenges need to be tackled in following clinical EVs application, such as the sources, large-scale production, standard of isolated process and purify, half-time degradation, administration route and frequency and dosage (Bruno et al., 2020b; Tang et al., 2020).

## Conclusion and Future Prospects

In this review, we highlighted the roles of EVs in cellular and organic communication. Most studies demonstrated that EVs exacerbated MAFLD development. Recently, it was found that in early obesity mice, hepatocytes secreted miR-3075-enriched EVs to improve insulin sensitivity. But in chronic obesity, this compensatory mechanism disappeared. The function of hepatocytes EVs was converted into promoting IR (Ji et al., 2021). This result suggested that EVs had different effects during different stages of MAFLD. It seemed to explain why some EVs showed a protective effect in previous studies. For example, some adipose tissue-derived EVs activated the insulin pathway of hepatocytes; plasma EVs in normal and obese people inhibited inflammation of hepatocytes.

Another research interest in MAFLD is immune response. Immune cells can serve as both donor cells and target cells of EVs. Most studies in EVs are about macrophages or BMDMs. Other immunocytes, such as T cells and neutrophils may also participate in the release and uptake of EVs. Together, they construct an immune network in innate and adaptive immunity. How EVs and their cargos regulate the immune network requires more investigation.

Thus far, MAFLD has no approved drug therapies. Previous researches showed that EVs were associated with the pathogenesis of MAFLD. In this context, impeding harmful functions of EVs may be new targets for clinical intervention. On the other side, there are some protective molecules existing in harmful EVs, which play a compensatory role. Elevating the levels of these molecules exogenously may relieve MAFLD. And for potential clinical use, stem cell-derived EVs may be safer and have fewer side effects. Compared with conventional drug delivery, EVs have superior advantages as secretory nanoparticles. So, manipulating EVs as a neo-drug delivery platform holds great promise for improving MAFLD progression.
